# Delineation of the trigeminal-lateral parabrachial-central amygdala tract in humans

**DOI:** 10.1162/imag_a_00567

**Published:** 2025-05-02

**Authors:** Batu Kaya, Omar Khalil, Spencer S. Abssy, Iacopo Cioffi, Massieh Moayedi

**Affiliations:** Centre for Multimodal Sensorimotor and Pain Research, Faculty of Dentistry, University of Toronto, Toronto, ON, Canada; Clinical & Computational Neuroscience, Krembil Research Institute, University Health Network, Toronto, ON, Canada; University of Toronto Centre for the Study of Pain, Toronto, ON, Canada; Department of Dentistry, Mount Sinai Hospital, Toronto, ON, Canada

**Keywords:** amygdala, diffusion MRI, tractography, ultra-high field imaging, brain, white matter, parabrachial nucleus, pain affect, pain unpleasantness, orofacial pain, structural MRI, connectivity

## Abstract

The parabrachial nucleus (PBN) relays interoceptive and exteroceptive information to limbic brain regions. In particular, lateral PBN projections to the central nucleus of the amygdala (CeA) are thought to encode the affective dimension of pain. Pain in the orofacial region is thought to be more unpleasant than pain elsewhere in the body because the face plays an important role in social interactions, feeding, and exploration. The trigeminal nerve (CN V) carries sensory, including nociceptive, information from the orofacial region to the central nervous system. Canonically, the affective dimension of orofacial pain is thought to be encoded by the medial trigeminothalamic tract, which projects to midline thalamic nuclei and further to limbic brain regions. A preclinical study identified an additional circuit that carries orofacial nociceptive information to subcortical limbic brain regions via the lateral PBN. This circuit, from the CN V to the lateral PBN and further to the CeA, is thought to contribute to the heightened negative affect of orofacial pain. However, the CN V–lateral PBN–CeA circuit has yet to be delineated in humans. Here, we aimed to resolve this circuit in humans with diffusion MRI from the Human Connectome Project (HCP) using probabilistic tractography. We first delineated the tract at 7T (n = 150) to determine whether this tract can be resolved. Next, we delineated the tract at the more readily available, but lower resolution 3T field strength (n = 155). Given the growing evidence of sex differences in pain mechanisms, as a secondary aim, we explored whether sex differences in connectivity strengths of the circuit existed in our sample. The basolateral amygdala (BLAT) was used as a negative control, as we did not anticipate CN V-lPBN-BLAT connectivity. The CN V–lPBN–CeA circuit had significantly stronger connectivity strength than the BLAT circuit at both field strengths (both*p*< 0.001). Only the right CN V–lPBN–CeA circuit at 3T showed significantly stronger connectivity in males than in females (*p*= 0.002). This study delineated the human CN V–lPBN–CeA circuit for the first time*in vivo.*This circuit may provide a neuroanatomical substrate for the heightened negative affect elicited by orofacial pain and could serve as a potential therapeutic target.

## 1 Introduction

The parabrachial nucleus (PBN) receives interoceptive and exteroceptive information and projects to limbic brain regions (Al-Khater & Todd, 2009;Bernard et al., 1994;Grigson et al., 1998;Li & Sheets, 2020;Raver et al., 2020;Wilson et al., 2019). Specifically, it relays nociceptive (Sun et al., 2020), pruritic (Mu et al., 2017), and gustatory (Fu et al., 2019) signals, and contributes to the development and maintenance of neuropathic pain (Uddin et al., 2018), aversive learning (Han et al., 2015), and feeding behaviours (Alhadeff et al., 2018), among others. Projections from the lateral parabrachial nucleus (lateral PBN) to the central nucleus of the amygdala (CeA) carry nociceptive information from the body and contribute to the affective dimension of pain.

Orofacial pain is thought to have a greater affective component (Auvray et al., 2010) and is more unpleasant than pain elsewhere on the body (Schmidt et al., 2015,^2016^;Zakrzewska et al., 2017). This is because the orofacial region has special biological, emotional, and psychological significance given its crucial roles in social, feeding, and exploratory behaviours (Sessle, 2000). However, the neural underpinnings of the heightened affective response to orofacial pain in humans remain unknown.

Nociceptive information from the orofacial region is carried by the trigeminal nerve (the 5^th^cranial nerve; CN V) to the thalamus via the trigeminothalamic tract (Sessle, 2019). Specifically, the dorsal trigeminothalamic tract carries nociceptive information from the orofacial region to limbic brain regions via midline thalamic nuclei (Terrier et al., 2022).

A preclinical study identified an additional monosynaptic circuit that carries orofacial nociceptive information to limbic brain regions (Rodriguez et al., 2017). This circuit, from the CN V to the lateral PBN (lPBN) and further to the CeA, contributes to the heightened negative affect elicited by orofacial pain in mice (Rodriguez et al., 2017).

It is thus feasible that the CN V–lPBN–CeA circuit contributes to the heightened orofacial pain affect in humans. However, the presence of this circuit in humans has yet to be established. To investigate this circuit in acute and chronic pain, it is essential to first determine whether it can be resolved using neuroimaging in humans.

Diffusion magnetic resonance imaging (dMRI) and tractography have been successfully used to non-invasively delineate complex white matter pathways in humans. Briefly, dMRI relies on anisotropic diffusion of water in neural tissues to infer the dominant orientations of white matter in each voxel. Tractography relies on these orientations to reconstruct white matter pathways connecting brain regions (Behrens et al., 2007;Dyrby et al., 2007;Parker, 2004). DMRI has been used to delineate previously undefined brain tracts—that is, hodology (Catani, 2007), at both 3T and 7T (Cormie et al., 2023;Kaya et al., 2023;Shim & Baek, 2022).

There is emerging evidence of sexually dimorphic neural mechanisms of nociception, pain-related brain circuits, and pain modulatory pathways (Cantu et al., 2022;Mogil et al., 2024;Osborne & Davis, 2022;Presto et al., 2022;Stratton et al., 2024). Furthermore, the prevalence of acute and chronic orofacial pain is greater in females than in males (Haggman-Henrikson et al., 2020;Sessle, 2021;Shinal & Fillingim, 2007). Therefore, should the presence of this circuit be established in humans, further investigation of sex differences is warranted.

The overall goal of this study was to determine whether the CN V–lPBN–CeA circuit can be resolved in humans using dMRI data. Given the small size of structures in the CN V–lPBN–CeA circuit, we first sought to delineate this circuit with ultra-high field (UHF; 7T) imaging. 7T imaging provides a higher signal-to-noise ratio, spatial resolution, and contrast-to-noise ratio than the more conventional and readily accessible 3T imaging (Sclocco et al., 2018), thus allowing for better visualization of smaller structures, such as those found in the brainstem. However, as 3T imaging is more established, we further sought to determine whether the circuit could be delineated at this lower field strength, as this would allow for future investigations in clinical populations, as well as investigations in extant data of orofacial pain. This approach has previously been used to delineate neural circuits at both 7T and 3T (Cormie et al., 2023;Kaya et al., 2023;Shim & Baek, 2022).

Here, we aimed to delineate the CN V–lPBN–CeA circuit*in vivo*using MRI data from the Human Connectome Project (HCP). As a secondary aim, we determined whether there are sex differences in CN V–lPBN–CeA connectivity strength at both 7T and 3T. We hypothesize that we will resolve the CN V–lPBN–CeA circuit at both 7T and 3T and that the circuit will have stronger structural connectivity in females than in males.

## 2 Methods

All procedures were approved by the University of Toronto’s Human Research Ethics Board (Protocol Number: 40458). The data used in this study are openly available in the HCP database athttps://db.humanconnectome.org, HCP S1200 Release (February 2017), which comprises MRI scans from 1,200 participants. A subset of 173 participants also underwent 7T scanning.

### 2.1 Participants

We aimed to delineate the CN V–lPBN–CeA circuit in all 173 healthy young adults with both 7T and 3T diffusion images included in the HCP database following the final S1200 release. HCP participant inclusion/exclusion criteria for recruitment are publicly available (Van Essen et al., 2013). Briefly, exclusion criteria comprised significant history of psychiatric disorders, substance use disorders, neurological or cardiovascular disease, epilepsy, any genetic disorder, neurodegenerative disease, traumatic brain injury, ongoing chemo- and/or immunotherapy, and MRI contraindications. Readers interested in the full exclusion criteria are encouraged to refer to supplemental table 1 inVan Essen et al. (2013).

Eight participants were excluded: did not have dMRI data at 7T (n = 2), registration failure (n = 5), and the CN V could not be visualized (n = 1). The final sample included 165 participants (101 females, 64 males). HCP only provides age range categories for participants (22–25, 26–30, 31–35, 36+ years of age). We provide a breakdown of participants in each category, stratified by sex (seeTable 1).

**Table 1.** Age distribution in the HCP S1200 release with both 7T and 3T dMRI and T1 data included in this study.

Age group (years)	Male	Female	Whole group
22–25	17	1	18
26–30	33	47	80
31–35	14	51	65
36+	0	2	2

### 2.2 HCP imaging parameters

All participants in this study underwent MRI scanning at both 3T and 7T. The 3T scans were acquired at the Washington University in St. Louis, using a customized Siemens 3T “Connectome Skyra,” with a standard 32-channel head coil and a body transmission coil. Participants were then flown to the Center for Magnetic Resonance, University of Minnesota for 7T scans acquired with a Siemens Magnetom scanner. A single-channel transmit/32-channel receive head coil (Nova Medical, Wilmington, DE, USA) was used.

Whole brain structural T1 scans were acquired at 3T using a 3D MPRAGE sequence: repetition time (TR) = 2,400 ms, echo time (TE) = 2.14 ms, field of view (FOV) = 224 × 224, and 0.7 mm isotropic voxels. 7T spin-echo EPI diffusion-weighted scans were collected over four runs with the following parameters: TR = 7,000 ms, TE = 71.2 ms, FOV = 210 × 210, 1.05 mm isotropic voxels, and 132 slices. These were acquired with two sets of gradient tables, each with two different*b*-values (b = 1,000 s/mm^2^and b = 2,000 s/mm^2^) interspersed with an equal number of acquisitions on each shell within each run. The uniform distribution of directions across shells was ensured with the Emmanuel Caruyer toolbox (Caruyer et al., 2013). Each set included 65 diffusion-weighted directions and 6 non-diffusion-weighted images (B0s) interspersed across each run. Two of the four acquisitions were performed once with each anterior-to-posterior and the other two with posterior-to-anterior encoding polarities. 3T spin-echo EPI diffusion-weighted scans were collected over six runs with the following parameters: TR = 5,520 ms, TE = 89.5 ms, FOV = 210 × 180, 1.25 mm isotropic voxels, and 111 slices. These were acquired with three sets of gradient tables, each with three different*b*-values (b = 1,000 s/mm^2^, b = 2,000 s/mm^2^, and b = 3,000 s/mm^2^) interspersed with an equal number of acquisitions on each shell within each run. The uniform distribution of directions across shells was ensured with the Emmanuel Caruyer toolbox. Each set included 90 diffusion-weighted directions and 6 B0s interspersed across each run. Three of the six acquisitions were performed once with each anterior-to-posterior and the other three with posterior-to-anterior encoding polarities. The 1,200 Subjects Data Release Reference Manual by HCP can be consulted for further details on data acquisition (https://www.humanconnectome.org/storage/app/media/documentation/s1200/HCP_S1200_Release_Reference_Manual.pdf).

### 2.3 MRI and statistical analysis

#### 2.3.1 HCP preprocessing

For each participant, we downloaded the preprocessed whole brain T1 scan (T1w_acpc_dc_restore.nii.gz) and the diffusion-weighted scans at 7T and 3T. The HCP pre-processing pipeline scripts are publicly available on Github (https://github.com/Washington-University/HCPpipelines). Details of the minimal preprocessing pipelines can be reviewed inGlasser et al. (2013).

Briefly, T1 images were corrected for gradient distortion and then aligned using a rigid-body alignment with 6 degrees of freedom (DOF) and averaged to create a single T1 image (if there was more than one run). The FMRIB software library (FSL, v 5.0.6;https://fsl.fmrib.ox.ac.uk/fsl) Linear Image Registration Tool (FLIRT) (Jenkinson et al., 2002,^2012^;Smith et al., 2004) was used to register the T1 image to the Montreal Neurological Institutes (MNI) standardized space template using 12 DOF.

For dMRIs, FSL was used to normalize B0 intensity using FSL’s Diffusion Toolbox (FDT) (Jenkinson et al., 2012), after which susceptibility-induced B0 field deviations were calculated. Eddy current distortion and participant motion were corrected using the eddy tool in FDT (Andersson & Sotiropoulos, 2016). After correcting for these deviations, the diffusion-weighted image was resampled into the participant’s native space using FLIRT. A binary brain mask was generated for fibre orientation estimation using FSL’s BEDPOSTx tool (Behrens et al., 2003,^2007^).

### 2.4 Study-specific preprocessing

We employed a liberal skull-stripping approach for dMRI scans to ensure the inclusion of the CN V in the resultant brain mask using FSL’s brain extraction tool (BET v6.0.3) (Jenkinson et al., 2005). Each skull-stripped B0 scan was quality controlled to ensure the inclusion of bilateral CN V. In cases where the full CN V was not included by BET, we manually adjusted the masks to reincorporate the CN V. T1-weighted scans were skull stripped using the SynthStrip toolbox (Hoopes et al., 2022) in FreeSurfer v7.3.2 (Fischl, 2012). DMRIs were aligned to T1 images with affine registration using FLIRT (Jenkinson et al., 2002). Linear transformations were used to align T1 images to the 1 mm MNI152 template. A concatenated matrix to register dMRI images to the MNI template and inverse registration matrices were computed. Probabilistic CeA, BLAT, PAG, and lPBN seeds were transformed from MNI space to diffusion space for each participant. These were visually inspected for anatomical accuracy. A tensor was fit using FSL’s DTIFIT to visualize major fibre orientations for the entire brain. CN V seeds and exclusion masks were manually drawn. See*MRI and Statistical Analysis: Probabilistic Tractography*for details. Two principal fibre directions were modelled for every voxel using BEDPOSTx. Lastly, we used Probtrackx (Behrens et al., 2007) to perform tractography and determine connectivity strength between our regions of interest (ROIs). The resulting tracts were resampled from diffusion space to MNI space for visualization purposes.

### 2.5 Regions of interest definition

**Fig. 1.**
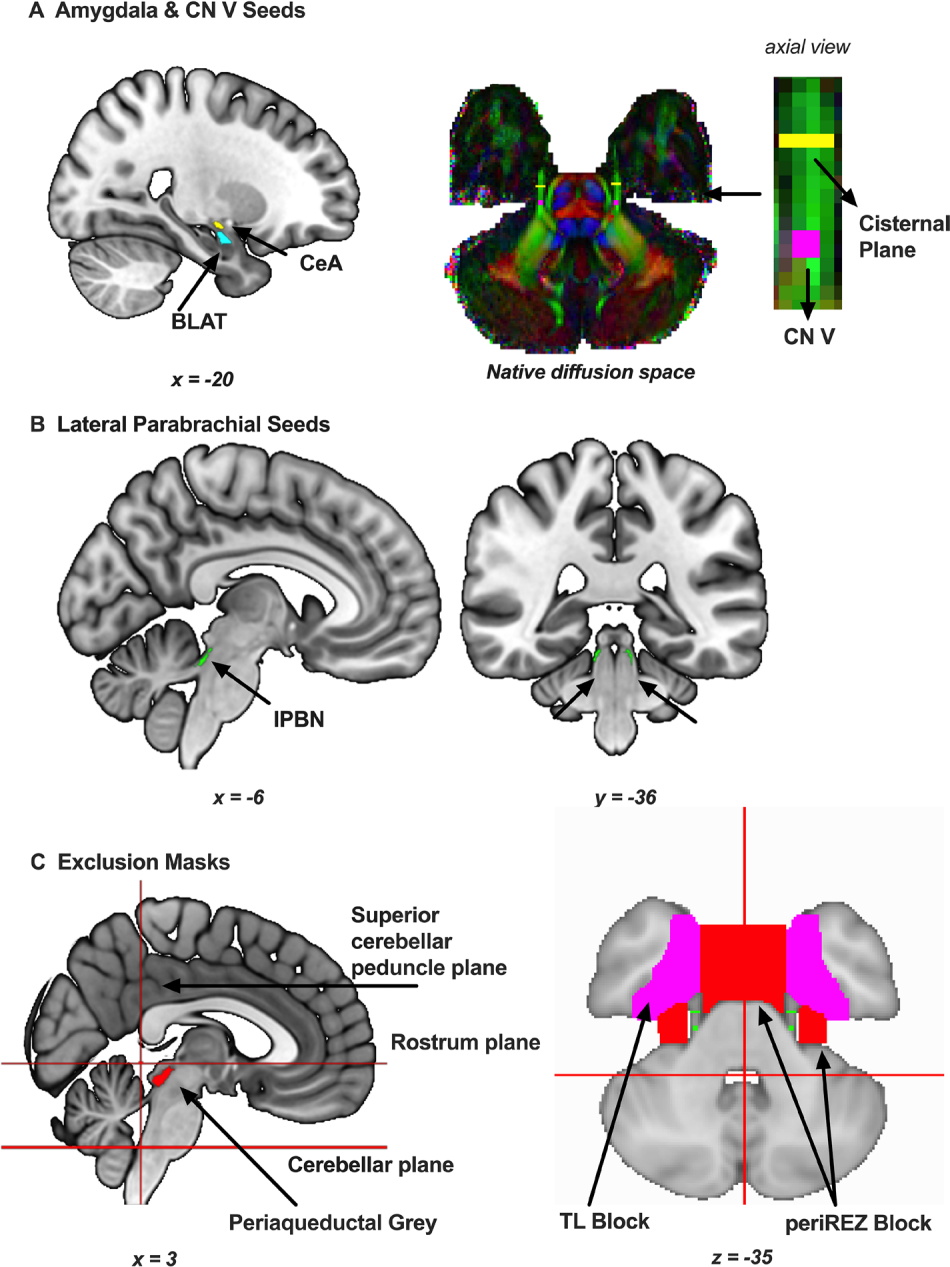
Seeds and exclusion masks used in tractography. (A) Central (in yellow) and basolateral (in teal) amygdala (CeA and BLAT, respectively) shown on an axial T1-weighted image in MNI152 space. The cisternal plane seed (in yellow) and root entry zone (in pink) for the trigeminal nerve (CN V) are shown in a participant’s native diffusion space. (B) Lateral parabrachial (lPBN) nucleus shown in green on a T1-weighted image in MNI152 space. (C) Exclusion masks used to constrain tractography are shown on a T1-weighted image in MNI152 space. Exclusion planes of the superior cerebellar peduncle and the cerebellum, and seeds of the periaqueductal gray and the region peripheral to the CN V root-entry zone (periREZ) are shown in red. An exclusion mask of ventrolateral aspect of the temporal lobe is shown in pink. TL: temporal lobe, periREZ: outside perimeter of the CN V root entry zone. MNI coordinates can be found below each brain slice.

Figure 1provides a schematic of the ROIs used in the study.

#### 2.5.1 CN V

The cisternal plane (CP) and the root entry zone (REZ) of the CN V were used as a proxy for the trigeminal ganglion to capture trigeminal afferents coursing to the central nervous system (Fig. 1A). Recent work shows that a two-ROI method is superior to both single-ROI and three-ROI methods for tracking the trigeminal nerve (Xie et al., 2020). Additionally, the REZ was used as a waypoint to ensure that samples were propagated towards the brainstem. The first eigenvector image produced by the tensor fit was modulated by fractional anisotropy values to visualize fibre direction with colour. The CN V was then located in the axial plane. A 2 x 2 x 2 cube (8 voxels, 9.261 mm^3^) was drawn at the CN V REZ. Then, a 1 x 4 x 4 CP ROI was drawn anterior to the REZ (16 voxels, 18.522 mm^3^). We located the widest fanning segment of CN V fibres anterior to the REZ and outside the temporal lobes when picking the location of CP. This process was performed bilaterally.

#### 2.5.2 Amygdala

TheTyszka and Pauli (2016)atlas provides probabilistic ROIs for amygdalar subnuclei in standard MNI space. This atlas is based on a sample of 168 healthy adults with an age range of 22–35 years from the HCP database. Two seeds, CeA and basolateral amygdala (BLAT), were used in probabilistic tractography as final targets and the origin when propagating putative tracts in an ascending, (i.e., brainstem to subcortex) and descending manner (i.e., subcortex to brainstem), respectively (Fig. 1A). Several naming conventions exist for the amygdalar basolateral complex (LeDoux, 2007). To avoid confusion, we followed the classifications used in this probabilistic atlas. As such, the BLAT seed in this study does not include the lateral and accessory basal/basomedial subnuclei. Additionally, we chose to remove the parvocellular division of the basolateral nucleus from the BLAT seed, since the developers of the atlas noted that this specific nucleus was merged into an unresolved nucleus (i.e., paralaminar) when creating the final seeds for their probabilistic atlas. In short, the BLAT seed in this study included the fully defined dorsal and intermediate divisions of the basolateral nucleus.

### 2.6 Lateral parabrachial nucleus & periaqueductal grey

The Brainstem Navigator atlas (v0.9;https://www.nitrc.org/projects/brainstemnavig/) provides probabilistic ROIs for brainstem subnuclei crucial for sensorimotor, arousal and autonomic functions in MNI space (Bianciardi et al., 2015). Semi-automatic and manual segmentations of these nuclei were carried out using 7T, multi-contrast (T2 and diffusion fractional anisotropy) MRI data from*in vivo*to generate the seeds. In this study, the 35% thresholded lPBN seeds were used as waypoints in probabilistic tractography (Fig. 1B). The probabilistic periaqueductal grey (PAG) ROI was thresholded at 50% to generate an exclusion mask (Fig. 1C). The difference in threshold values between the seeds is related to the size and location of these brain regions. The lPBN comprises a relatively small patch of cells located at the boundary between the superior cerebellar peduncle and the cerebral aqueduct—an area prone to imaging distortions. Thus, we employed a liberal threshold to ensure that the ROI would capture the lPBN in each individual after transformation to individual space. Given that the PAG ROI is used as an exclusion mask, we employed a more conservative threshold to reduce the risk of false negatives. The specific atlas labels for the lPBN seeds in this atlas are LPB-l and LPB-r, and PAG for the PAG seed. All amygdalar and brainstem ROIs were binarized.

### 2.7 Probabilistic tractography

We performed probabilistic tractography to assess CN V–lPBN–CeA connectivity. The CN V–lPBN–BLAT served as a control circuit since CeA is the only amygdalar subnucleus with dense connectivity with the brainstem (McDonald, 2020). Each participant had a total of eight tractograms: within one hemisphere, fibres were constructed in both directions (i.e., Origin-to-Target; Target-to-Origin) for each circuit to account for directional biases during acquisition (Van Essen et al., 2014) and fibre fanning (Jeurissen et al., 2019). This resulted in four tractograms per hemisphere. We used the modified Euler algorithm and set the number of samples to 10,000. A curvature threshold of 0.1 was used to allow for sharper turns for paths crossing from brainstem to subcortical regions. Path distribution was corrected for the length of the pathways.

Tract propagation was further refined using exclusion planes to limit the boundaries of the path distribution space in the brain, and by incorporating waypoints as relays that had to be crossed to reach a target ( Fig. 1C ). Specifically:

1. A midline mask was drawn in diffusion space to constrain tracts to a single hemisphere.
2. An axial plane below the rostrum of the corpus callosum was drawn in diffusion space to discard projections from CeA to the neocortex.
3. A coronal plane across the superior cerebellar peduncle was drawn in diffusion space to discard cerebellar fibres. We ensured that there was no contiguity between the lPBN seeds and this exclusion plane for each participant.
4. An axial pontine plane was drawn above the medulla oblongata in diffusion space to remove pyramidal and spinothalamic fibres.
5. Due to PAG’s extensive projections to the lPBN (Krout et al., 1998), we added the PAG seed as an exclusion seed while propagating tracts.
6. An exclusion mask was manually drawn in diffusion space to fill in the areas containing cerebrospinal fluid in Meckel’s cave. This “periREZ” seed ensured that the propagated fibres would not traverse across anatomically implausible locations at the brainstem–cerebrospinal fluid boundaries. We ensured that there was no contiguity between the trigeminal and waypoint seeds and the periREZ seed for each participant.
7. An exclusion mask of the ventromedial aspect of the temporal lobe (i.e., the parahippocampal gyrus and the temporal pole; TL block) was manually drawn on the same axial slices where the periREZ seed was present. The TLBlock was used to ensure that putative tracts followed anatomically plausible trajectories when ascending and descending towards their target. We effectively constrained the trajectory of the tracts to the brainstem–subcortex axis by blocking the anatomically implausible tract propagation directly to the cortex, without first traversing across the brainstem and the subcortex.
8. A probabilistic mask of the CN V motor nucleus from a previous study (Kaya et al., 2023) was thresholded at 50% and used as an exclusion mask to discard pontine CN V fibres (Guberinic et al., 2020).
9. To ensure that tracts did not propagate past CeA or CP depending on the direction in which fibres were propagated, we set the target seed both as a waypoint and a termination mask.

Additionally, REZ and lPBN seeds were included as waypoint masks independent of directionality. Ascending tracts were propagated from CP towards REZ, then lPBN, and eventually to their amygdalar target. Tracts in the opposing direction originated in the amygdala ROI (CeA or BLAT) propagated to the lPBN, then the REZ, and terminated at CP.

### 2.8 Statistical analyses

We quantified the connectivity strength for the circuits in the study using the “waytotal count” metric generated by the probabilistic tractography algorithm. This count is equal to the number of streamlines that reach the final target seed in accordance with pre-defined user criteria (see section*Probabilistic Tractography*for more details). Initially, the total number of streamlines is equal to the product of the number of voxels in the seed of origin and the number of total streamlines to be used in propagation, which was set to 10,000 in this study. However, given the differences between the number of voxels in the trigeminal and amygdalar seeds, we normalized the number of streamlines that reached the target by dividing the waytotal count by the number of voxels in the initial seed. These normalized waytotal counts between the same origin and target seeds (e.g., Right CN V to Right CeA, Right CeA to Right CN V) were averaged. In the end, there were four probabilistic connectivity strength measurements, that is, waytotal counts, per participant: Right CN V–lPBN–CeA, Right CN V–lPBN–BLAT, Left CN V–lPBN–CeA, and Left CN V–lPBN–BLAT. Shapiro–Wilk tests were run to assess normality (*p*< 0.05). Given that data were not normally distributed, we performed Wilcoxon signed-rank tests to compare the connectivity strength of the CN V–lPBN–CeA and CN V–lPBN–BLAT circuits in each hemisphere. We used a Bonferroni-adjusted alpha of 0.025 since two statistical comparisons were run within the same magnetic field strength (i.e., right vs. left hemisphere in both 7T and 3T).

We then grouped our data in a sex-disaggregated manner, that is, males versus females, at 7T and 3T. Shapiro–Wilk tests were run to assess normality (*p*< 0.05). Given that data were non-normally distributed, we computed Wilcoxon signed-rank tests to determine whether the CN V–lPBN–CeA circuit had stronger connectivity strength than the control circuit within each sex. We used a Bonferroni-adjusted alpha of*p*= 0.025 since two statistical comparisons were run within the same magnetic field strength. Lastly, we computed four Mann–Whitney U tests—two per magnetic field strength, to test for sex differences in the connectivity strength of the CN V–lPBN–CeA circuit.

## 3 Results

### 3.1 Outliers and normality testing

Data points 3 standard deviations above the mean were removed prior to statistical analyses. After outlier removal, final sample sizes were 150 (56 M, 94 F) and 155 (59 M, 96 F) at 7T and 3T, respectively. For brevity, the CN V–lPBN–CeA circuit and the CN V–lPBN–BLAT circuit are referred to as the CeA circuit and the BLAT circuit, respectively, in the results section.

At 7T, connectivity strengths for both circuits diverged significantly from a normal distribution in both hemispheres; Right CeA_7T_:*S-W*_150_= 0.720,*p*< 0.001; Right BLAT_7T_:*S-W*_150_= 0.713,*p*< 0.001; Left CeA_7T_:*S-W*_150_= 0.681,*p*< 0.001; Left BLAT_7T_:*S-W*_150_= 0.674,*p*< 0.001. Similarly, at 3T connectivity strengths for both circuits diverged significantly from normal distribution in both hemispheres; Right CeA_3T_:*S-W*_155_= 0.667,*p*< 0.001; Right BLAT_3T_:*S-W*_155_= 0.725,*p*< 0.001; Left CeA_3T_:*S-W*_155_= 0.742,*p*< 0.001; Left BLAT_3T_:*S-W*_155_= 0.649,*p*< 0.001.

### 3.2 Connectivity strength differences between the CeA circuit and the BLAT circuit

**Fig. 2.**
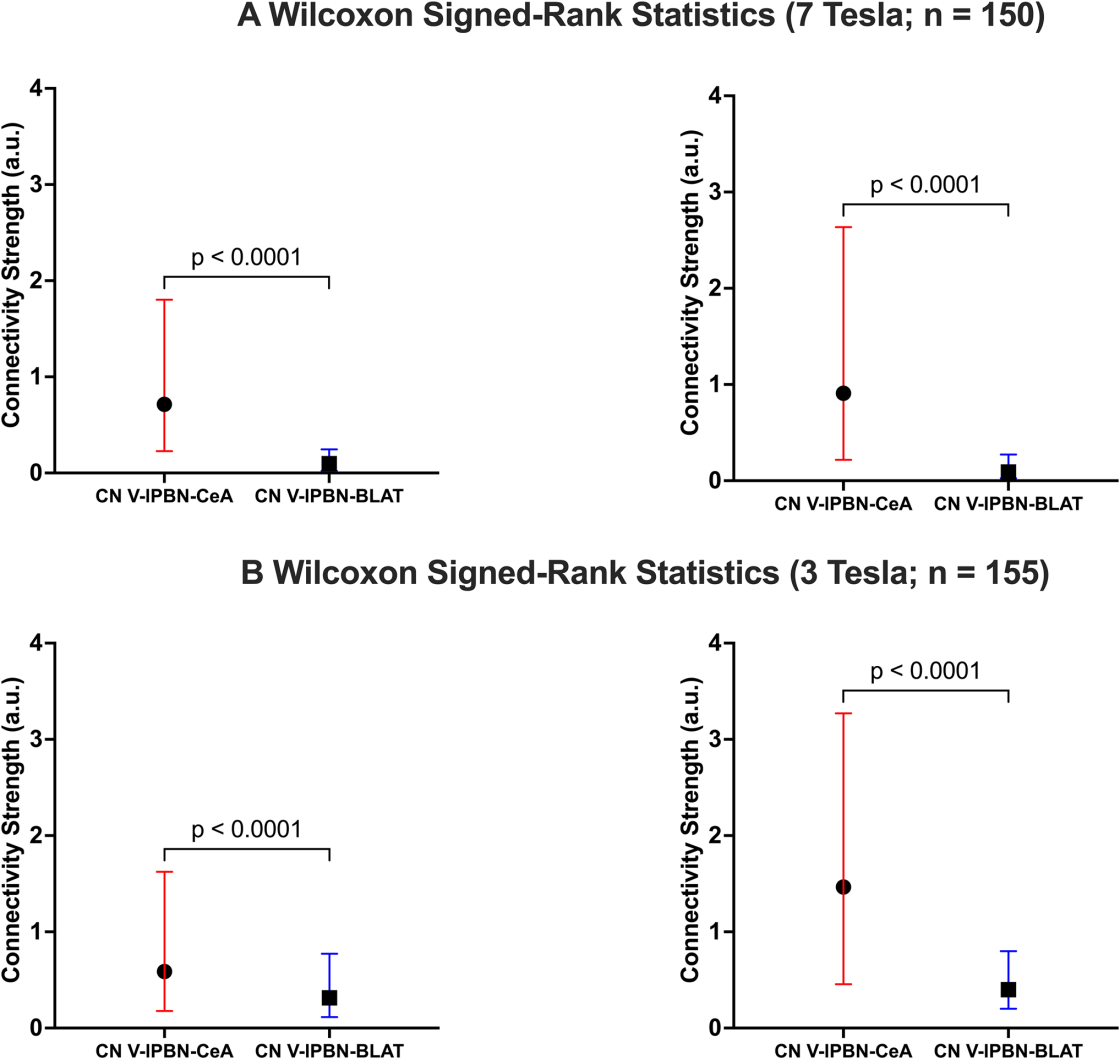
The CN V–lPBN–CeA circuit has stronger connectivity than the CN V–lPBN–BLAT circuit at both 7T and 3T. Median and the interquartile ranges for both circuits in the right and left hemispheres at (A) 7T and (B) 3T. Wilcoxon signed-rank comparisons reveal that in both hemispheres and for each field strength, the CN V–lPBN–CeA circuit has stronger connectivity than the CN V–lPBN–BLAT circuit.

At 7T, Wilcoxon signed-rank tests revealed stronger connectivity for the CeA circuit, than for the control BLAT circuit in both hemispheres (Right CeA_7T_vs. Right BLAT_7T_: W = 10,060,*p*< 0.001; Left CeA_7T_vs. Left BLAT_7T_: W = 10,132,*p*< 0.001;Fig. 2A). The median (±Interquartile range; IQR) connectivity strength for each circuit were Right CeA_7T_= 0.713 (1.51), Right BLAT_7T_= 0.0976 (0.221), Left CeA_7T_= 0.909 (2.39), Left BLAT_7T_= 0.0903 (0.239).

At 3T, Wilcoxon signed-rank tests revealed that the CeA circuit had stronger connectivity than the control BLAT circuit in both hemispheres (Right CeA_3T_vs. Right BLAT_3T_: W = 8,411,*p*< 0.001; Left CeA_3T_vs. Left BLAT_3T_: W = 9,994,*p*< 0.001;Fig. 2B). The median (±IQR) connectivity strength for each circuit were Right CeA_3T_= 0.588 (1.43), Right BLAT_3T_= 0.314 (0.654), Left CeA_3T_= 1.465 (2.80), Left BLAT_3T_= 0.399 (0.598).

### 3.3 Sex differences in connectivity strength

#### 3.3.1 Sex-disaggregated comparisons

We first assessed whether the data were normally distributed within each sex. At 7T, connectivity strengths for all four circuits diverged significantly from normal distribution in females: Right CeA_7T_:*S-W*_94_= 0.678,*p*< 0.001; Right BLAT_7T_:*S-W*_94_= 0.729,*p*< 0.001; Left CeA_7T_:*S-W*_94_= 0.708,*p*< 0.001; Left BLAT_7T_:*S-W*_94_= 0.762,*p*< 0.001; and in males: Right CeA_7T_:*S-W*_56_= 0.778,*p*< 0.001; Right BLAT_7T_:*S-W*_56_= 0.748,*p*< 0.001; Left CeA_7T_:*S-W*_56_= 0.633,*p*< 0.001; Left BLAT_7T_:*S-W*_56_= 0.620,*p*< 0.001.

We then compared the connectivity strength of each circuit within each sex using the non-parametric Wilcoxon rank test, with significance set at a Bonferroni-corrected alpha of 0.025 (0.05/2). In females, the CeA circuit had stronger connectivity than the BLAT circuit in both hemispheres (right: W = 3,798,*p*< 0.001; left: W = 4,003,*p*< 0.001. We observed the same results in males (right: W = 1,491,*p*< 0.001; left: W = 1,415,*p*< 0.001). Therefore, the CeA circuit had stronger connectivity than the control circuit, both at the whole-group level and within each sex at 7T.

We then compared the connectivity strength of the CeA circuit between sexes with a Mann–Whitney U test, with significance set to alpha = 0.025. We found no sex differences in the connectivity strength of this circuit at 7T: Right CeA_M_vs Right CeA_F_: U = 2,203,*p*= 0.095; and Left CeA_M_vs Left CeA_F_: U = 2,545,*p*= 0.735.

We repeated these same analyses at 3T. The connectivity strength of all four circuits were non-normally distributed in females: Right CeA_3T_:*S-W*_96_= 0.616,*p*< 0.001; Right BLAT_3T_:*S-W*_96_= 0.688,*p*< 0.001; Left CeA_3T_:*S-W*_96_= 0.719,*p*< 0.001; Left BLAT_3T_:*S-W*_96_= 0.652,*p*< 0.001; and in males: Right CeA_3T_:*S-W*_59_= 0.710,*p*< 0.001; Right BLAT_3T_:*S-W*_59_= 0.833,*p*< 0.001; Left CeA_3T_:*S-W*_59_= 0.767,*p*< 0.001; Left BLAT_3T_:*S-W*_59_= 0.737,*p*< 0.001.

Next, we compared the connectivity strength of each circuit within each sex with a Wilcoxon rank test. Significance was set at a Bonferroni-corrected alpha of 0.025 (0.05/2). In females, the CeA circuit showed stronger connectivity than the BLAT circuit in both hemispheres (Right: W = 2,944,*p*= 0.014; Left: W = 3,811,*p*< 0.001). In males, the CeA circuit showed stronger connectivity than the BLAT circuit in both hemispheres (Right: W = 1,393,*p*< 0.001; Left: W = 1,486,*p*< 0.001). Therefore, the CeA circuit had significantly stronger connectivity than the BLAT circuit at the whole-group level and within each sex at 3T.

Finally, we compared the connectivity strength of the CeA circuit between the sexes with a Mann–Whitney U test with alpha = 0.025. There were no sex differences in the connectivity strength of this circuit in the left hemisphere: U = 2,827,*p*= 0.985. The right CeA circuit had stronger connectivity in males than in females with U = 1,987,*p*= 0.002. In males, the median (±IQR) connectivity strength of this circuit was 1.07 (2.47), whereas for females the median (±IQR) connectivity strength was 0.375 (1.04).

### 3.4 Visualizing the CN V–lPBN–CeA circuit

We thresholded each participant’s circuit tractograms prior to concatenating these across participants to visualize the circuit at the group level. Here, origin-to-target circuit tractograms were thresholded by the number of voxels in the origin seed multiplied by the number of samples per voxel (i.e., 10,000) at 0.2%. Then, the images for both directions of tractography were averaged, binarized, and resampled to standard space to collate them across participants. This process resulted in four final tractograms per field strength: Right CeA, Left CeA, Right BLAT, and Left BLAT. These group tractograms do not include data from participants with waytotal counts that were outliers.

**Fig. 3.**
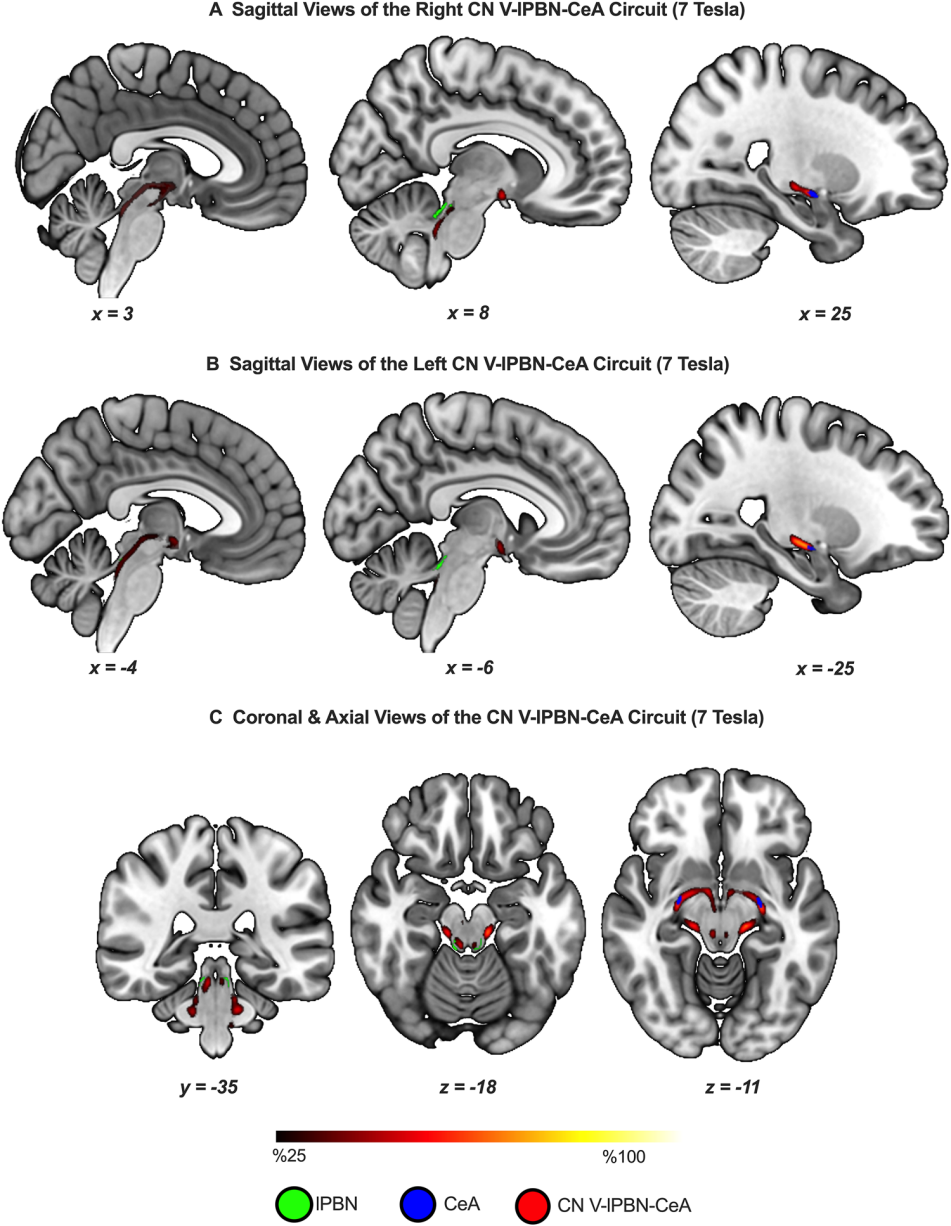
The CN V–lPBN–CeA can be delineated*in vivo*using ultra-high field 7T images. Group tractograms are based on N = 150, thresholded to show at least 25% overlap across participants. (A) and (B) show the right and left circuits, respectively. (C) illustrates passage of the tracts through the lateral parabrachial nuclei and termination in CeA in concordance with the waypoint and termination specifications in the probabilistic tractography algorithm, respectively. The group tractograms were resampled from diffusion space to the standard MNI space using a linear transform.

**Fig. 4.**
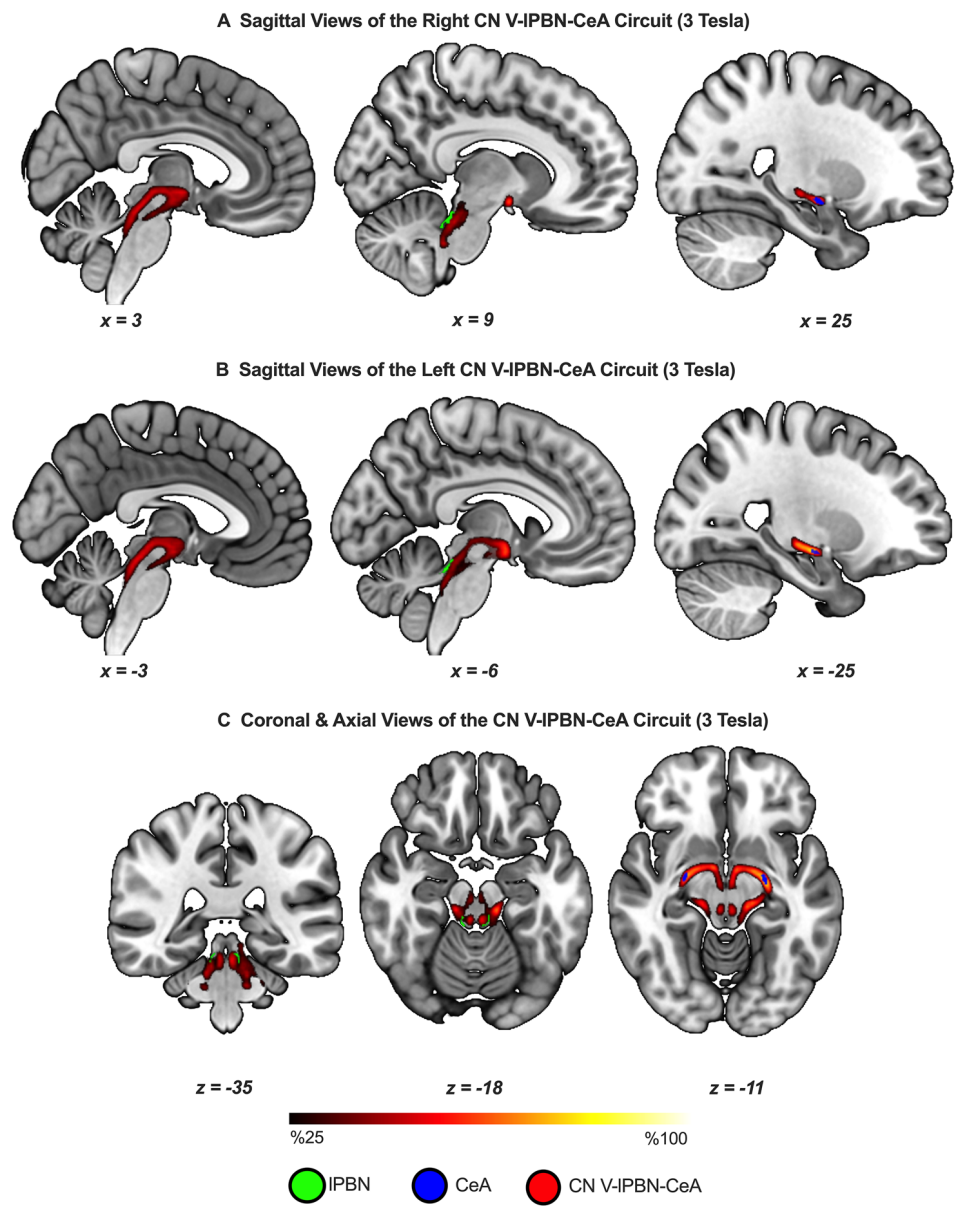
The CN V–lPBN–CeA can be delineated*in vivo*using 3T images. Group tractograms are based on N = 155, thresholded to show at least 25% overlap across participants. (A) and (B) show the right and left circuits, respectively. (C) illustrates passage of the tracts through the lateral parabrachial nuclei and termination in CeA in concordance with the waypoint and termination specifications in the probabilistic tractography algorithm, respectively. The group tractograms were resampled from diffusion space to the standard MNI space using a linear transform.

We successfully delineated and visualized the CeA circuit in both hemispheres at 7T (Fig. 3) and 3T (Fig. 4).

## 4 Discussion

In this study, we successfully delineated for the first time the CN V–lPBN–CeA pathway in humans at 7T. We then repeated this at the more readily available 3T field strength. Furthermore, given emerging evidence of sexually dimorphic pain mechanisms, we tested for sex differences in connectivity strength of the circuit. We did not identify sex differences at 7T but did find that the right CN V–lPBN–CeA pathway had significantly stronger connectivity in males than in females at 3T.

Preclinical models have significantly advanced our understanding of the neural basis of behaviour (Galvan et al., 2018;Vaidya et al., 2019). Methods such as retro/anterograde labelling, optogenetics, and chemogenetics require that neural tissue be probed invasively. Although preclinical trials are proposed to introduce the first-ever use of some of these methods in the human nervous system, regulatory and financial challenges remain significant, and vectors still need to be introduced invasively (Bansal et al., 2023;Claes et al., 2022;White et al., 2020). However, not all neural circuits are maintained across species. Therefore, it is essential to determine whether circuits and mechanisms identified in animal models can be resolved in humans. Non-invasive imaging approaches provide an opportunity to safely investigate the neural architecture of the human connectome*in vivo*. The proliferation of open-source databases such as the Human Connectome Project (HCP) (Van Essen et al., 2013) and probabilistic atlases for brain regions (Cauzzo et al., 2022;Singh et al., 2020;Tang et al., 2018;Tyszka & Pauli, 2016) allow us to construct putative white matter circuits informed by findings in the preclinical literature.

In the current study, we utilized significant increases in image resolution in ultra-high field (UHF) 7T dMRI from the HCP to counteract constraints inherent to brainstem imaging and probabilistic tractography such as weak contrast between white and grey matter (Sclocco et al., 2018) and fibre-fanning (Jeurissen et al., 2019), respectively. Although 7T machines are becoming more available, 3T scanners remain significantly more accessible and cost-effective (Ladd, 2007). The main benefits of UHF imaging are a higher signal-to-noise ratio and higher spatial resolution than 3T, allowing for better visualization of smaller structures and an increased ability to differentiate different tissue types (Sclocco et al., 2018). These two advantages are integral to imaging brainstem nuclei. However, UHF imaging is prone to susceptibility-induced geometric distortions and reduces signal uniformity (Jones et al., 2021).

The successful delineation of white matter circuits with dMRI at 7T can then be replicated in the more conventionally available 3T field strength, allowing for more research on their structural and functional features. This approach was used to delineate connectivity within the basal ganglia (Shim & Baek, 2022), insula–cingulate cortex projections (Cormie et al., 2023), and trigeminal circuits (Kaya et al., 2023). We note that statistical comparisons between the two field strengths should be interpreted with caution due to differences in acquisition parameters (e.g., voxel size, number of diffusion-weighted directions—a limitation of the HCP dataset). Nonetheless, these studies report overall qualitative and, when applicable, quantitative agreement between the delineated tracts at 3T and 7T. In the current study, we delineated the CN V–lPBN–CeA circuit in the more conventionally available 3T field strength, showing that UHF imaging is not necessary to delineate the tract. The current proof-of-concept study highlights the possibility of investigating the functional role of this circuit in the affective dimension of pain in humans at both 7T and 3T.

The CN V–lPBN–CeA circuit is thought to contribute to the heightened affective response elicited by orofacial pain. Specifically, the amygdala is responsible for assigning an emotional value to environmental stressors (LeDoux, 2007). In the context of pain, the functional role of the amygdala is thought to be related to enhanced suffering, fear of pain, and the initiation of motivational drive to mount nocifensive behaviours (Neugebauer, 2015). The CeA projects to several brainstem nuclei to modulate autonomic and motor responses to stimuli (Pitkänen et al., 1997). The capsular and lateral subnuclei of CeA are dubbed the “nociceptive amygdala” due to the direct excitatory inputs from the parabrachial nucleus (PB) (Allen et al., 2021). Similar to the amygdala, the lPBN plays an integrative role in modulating behavioural outputs in response to internal and external stressors, such as pain (Chiang et al., 2019). The extensive lPBN connections to CeA have been shown to be critical for avoidance and aversive memory in response to noxious stimulation in rodents (Chiang et al., 2020;Gauriau & Bernard, 2002;Neugebauer, 2015;Neugebauer et al., 2004;Palmiter, 2018). An inhibitory CeA–PB pathway has been proposed to modulate pain behaviours in tandem with the excitatory lPBN–CeA connections in rats and mice (Raver et al., 2020). Here, optogenetic activation of the inhibitory CeA–PB pathway was associated with a decrease in facial grimaces—a proxy for negative pain affect in rodents (Langford et al., 2010). Preclinical work has, therefore, outlined the critical involvement of the CeA–PB circuity in modulating affective responses to noxious input. However, the source of such noxious inputs might be just as crucial as the two brain regions mentioned.

There is substantial neuroanatomical evidence indicating that pain from the face versus the rest of the body is processed differently in the lPBN. In rodents, lPBN receives polymodal nociceptive input from lamina 1 neurons in the dorsal horn (Craig, 1995;Hermanson & Blomqvist, 1996). In comparison, orofacial nociceptive information gets transmitted via the trigeminal brainstem sensory nuclear complex (VBSNC). The VBSNC comprises the trigeminal main sensory nucleus, as well as the spinal trigeminal nucleus, which is made up of three subnuclei: oralis, interpolaris, and caudalis (Sessle, 2000). The entire complex receives noxious input from the periphery but the caudalis contains two overlapping somatotopic maps of the orofacial region (Dejerine, 1926). This organization is unique and might contribute to the enhanced importance of the orofacial region in psychosocial functions. Second-order afferents emerge from the VBSNC and project further to other brain regions. Although spinal and trigeminal nociceptive input overlap partially in PBN, in the lPBN these show anatomically segregated distributions (Slugg & Light, 1994), suggesting that bodily and orofacial pain are processed differentially here—much like in the ventrolateral thalamus. Tracing studies in rats show that the CeA and the PBN are connected via the spino (trigemino) –PBN–CeA circuit (Bernard et al., 1989;Jasmin et al., 1997;Ma & Peschanski, 1988). However, the neural circuity underlying enhanced affective responses to orofacial pain—as opposed to bodily pain—has remained an outstanding question.

Rodriguez et al. (2017)identified a direct monosynaptic circuit from the trigeminal ganglion to the external lPBN subnucleus, which bypasses the canonical VBSNC relay in mice. The authors then showed that awake-behaving mice emitted increased distress vocalizations, showed place preference in analgesic environments, and showed robust avoidance behaviours under optogenetic activation of the circuit—all indicators of negative affect. Another rodent study showed that external lPBN–CeA connectivity underlies the formation of aversive memory during Pavlovian threat conditioning (Han et al., 2015). Mice with the presynaptic functional inactivation of the lPBN–CeA circuit had intact responses to the sensory-discriminative components of pain, that is, hind paw lick latency. In contrast, nocifensive behaviours (i.e., escape jumping) were abolished. These results suggest that the spino(trigemino)–PBN–CeA pathway modulates affective responses to noxious stimuli in mice. However, it is unclear whether orofacial pain is indeed associated with enhanced negative affect in humans.

Few studies have investigated whether and how orofacial nociceptive input is processed differently than spinal nociceptive input in humans.Sambo, Liang et al. (2012)conducted two studies on the hand-blink reflex (HBR) to characterize the properties of the defensive peripersonal space (DPPS) surrounding the face. They first showed that the HBR had shorter onset latency, longer duration, and higher magnitude when the affected hand entered facial DPPS than when the hand was outside the DPPS (Sambo, Liang et al., 2012). These findings were later reproduced byBufacchi (2017),Bufacchi and Iannetti (2016), andBufacchi et al. (2016). In a follow-up study, they showed attenuation of this effect when a screen was placed between the hand and the face, which was accompanied by a significant decrease in perceived threat ratings for the screen versus no-screen conditions (Sambo, Forster et al., 2012). This suggests that the screen either interrupted the integration of visual and tactile input or, as posited by the authors, acted as a defensive shield against threats to the orofacial region. Finally, they showed that trigeminal neuralgia, a unilateral neuropathic pain disorder affecting the orofacial region, is associated with a unilateral expansion of the peripersonal space (Bufacchi, 2017;Bufacchi et al., 2017). Together, these data show that there is a unique DPPS for the orofacial region that can identify and integrate threats of potentially harmful stimuli towards it.

Further evidence for differential processing of noxious stimuli from the orofacial region comes from two studies that found that noxious heat delivered to the forehead was associated with higher fear of pain ratings and significantly higher within-subject sensitization than the identical stimulus delivered to the volar forearm (Schmidt et al., 2015). In a different study, capsaicin-induced hyperalgesia applied to the face elicited increased ipsilateral amygdalar activity in response to thermal and brush stimuli on the sensitized side compared with the control side (Moulton et al., 2007). Memory encoding for images associated with facial versus hand pain showed increased functional connectivity between the left amygdala and the visual processing, memory encoding, and object recognition areas (Schmidt et al., 2016,^2019^). The authors speculated that these effects were due to higher fear elicited by the aversiveness and threat of facial pain. Considered together, these findings suggest an enhanced amygdalar response to orofacial pain illustrated by increased physiological, functional connectivity, and behavioural metrics in healthy individuals.

Chronic orofacial pain disorders disproportionately affect females, and given the enhanced negative effect, they may be at higher risk of greater suffering. (Shinal & Fillingim, 2007). Furthermore, emerging evidence indicates that there may be sex differences in the neural mechanisms of pain (Mogil et al., 2024;Osborne & Davis, 2022;Stratton et al., 2024). As such, we investigated whether there were sex differences in the connectivity strengths of the CN V–lPBN–CeA circuits at both 7T and 3T. We hypothesized that the circuit would have stronger connectivity in females than in males. We did not find sex differences at 7T. At 3T, however, the right CN V–lPBN–CeA circuit showed significantly stronger connectivity in males than in females. We cannot draw any meaningful conclusions about these differences, given that 7T and 3T images had different acquisition parameters. As such, we could not compute statistical comparisons between the two different field strengths.Rodriguez et al. (2017)used both females and males in their study but did not report sex differences. Therefore, the evidence for a potential sex difference in the connectivity strength of this circuit remains inconclusive.

### 4.1 Limitations and future work

Human neuroimaging studies are inherently coarser than rodent tracing studies. In the current study, tractography was carried out using the entire CeA, as the subnuclei cannot be resolved; whereas animal literature indicates that the capsular and lateral, but not the medial, subnuclei receive lPBN inputs. Similarly, we used an lPBN seed, whereas theRodriguez et al. (2017)study found the external lPBN to be the region that relays orofacial nociceptive information to the CeA. To our knowledge, no probabilistic atlases to date provide seeds for subnuclei of these two regions, and it is not clear whether these could be reliably resolved with UHF MRI. We chose to assess unilateral direct CN V–lPBN–CeA connectivity in consideration of the rodent literature. Given that the spino-parabrachial tract comprised both ipsilateral and contralateral efferents from lPBN (Hermanson & Blomqvist, 1996;Slugg & Light, 1994), whether the novel CN V–lPBN–CeA circuit in humans has a contralateral component remains an outstanding question. This is likely, given the bilateral projections of trigeminal thalamic tracts (Henssen et al., 2016,^2019^). Although the ipsi- and contralateral components of the spino-parabrachial pathway have been shown to subserve different nociceptive processes in rodents at the spinal level (Deng et al., 2020), whether such differences are found for the trigeminal system has yet to be explored.

Our findings rely heavily on the definition of the ROIs used to delineate the CN V–lPBN–CeA circuit. Therefore, the choice of the atlas was carefully considered. For example, there are multiple ways to delineate amygdalar subnuclei. Automated segmentation methods, such as the amygdala parcellation in FreeSurfer, use algorithms to delineate amygdalar subnuclei based on templates (Saygin et al., 2017). Recent work shows that automated segmentation of amygdalar subnuclei is unreliable and lacks specificity (Kahhale et al., 2023). We opted to use the Tyszka–Pauli atlas as it was developed with the HCP 7T dataset and was delineated by neuroanatomical experts using the largest cohort of participants (n = 168) to create a probabilistic atlas of amygdala nuclei. Similarly, we used the Brainstem Navigator to derive lPBN and PAG ROIs for this study. Brainstem nuclei are very challenging to image due to their small size, the density of the number of nuclei in the brainstem region, and physiological noise (Sclocco et al., 2018). The Brainstem Imaging Laboratory addressed these challenges by obtaining ultra-high field 7T imaging data, manually delineating ROIs in these data, and validating the delineations*in vivo*and, for some nuclei,*ex vivo*. To date, the Brainstem Navigator atlas is the only publicly available atlas that has a mask for the lateral parabrachial nucleus. We encourage future studies to replicate our findings using different atlases and parcellation schemes.

Due to our inability to directly compare connectivity strength at 7T and 3T, we are not able to comment on potential sex differences in this circuit conclusively. We encourage future studies to investigate whether structural and/or functional differences can be identified across the CN V–lPBN–CeA circuit.

## 5 Conclusion

Rodent work in the last decade has provided specific neural circuity undergirding the affective dimension of nociceptive processing in the orofacial region. We provide the successful resolution of the CN V–lPBN–CeA white matter circuit*in vivo*for the first time at both 7T and 3T in humans. Future work involving human subjects can now investigate structural and functional differences along this pathway in experimental and clinical populations to further elucidate enhanced affect in response to orofacial pain.

## Data Availability

The data that support the findings of this study are openly available in the Human Connectome Project database athttps://db.humanconnectome.org, HCP S1200 Release (February 2017). Code used in the study pipeline is publicly available athttps://github.com/b2kaya/cnv-lpbn-cea.
